# Annual Meteorological Table for Sidmouth

**Published:** 1813-04

**Authors:** 


					Dr. Clarke's Meteorological Table for Sidmouth. 291
For the Medical and Physical Journal.
ANNUAL METEOROLOGICAL TABLE for SlDMOUTH,
Df.von. Lat. 50? 41' N. Lon. 3? 13' W.
By Dr. Cf.AiiKE.
1812.
THERMOMETER, NORTH EXPOSURE.
BA ROMETER.
January
Fc brun ry
March
April
May
June
July
August
Septetn.
October
Novem.
Decern, j
*19?i
53
55
57
66
71
75
76
70
65
67
54
sw
w
w
w
sw
N
SW
sw
sw
sw
s
s .
20?
28
22
26
36
37
36
40
34
31
24
23
NE
IN W I
NE
NE |
S
NE|
SE
NW]
INWI
W
N
NE
1^
42?|
48
46
52
59
04
67
67
66
53
48
39
31? |
36
34
36
44
43
45
46
40
40
36
31
o
37?|
42
39
44
51
54
56
57
53
49
42
35
g S
10?|
12
10
8
6
8
13
9
9
14
30.39
30.13
30.41
30.31
30.35
30.51
30.51
30.30
30.40
30.09
30.42
30.61
N
W
NW
NW
S
SE
N
SW
NW
SW
N
,NE
29.05
29.09
28.91
29.57
29.66
29.50
29.68
29.73
29.72
28.81
29.08
29.00
SW
S
SE
SW
S
SW
NW
SW
sw
sw
N
NW
29.96
29.64
29.78
29 97
29.93
30.03
30.06
30.07
30.13
29.57
29.99
30.00
a gj
^ ?
.72
.70
.92
.31
.41
.48
.33
.22
.28
.63
.42
.43
|feL
13
7
10
22
14
15
12
21
23
11
13
! 18
9
1.91
161
8
15
13
16
8
5
16
11
7
Thermometer.
Highest . . August 19, 76?
Lowest . . January 23, 20
Greatest ? October 7,3, 14
Variation $ '
Mean for the Day . , 55
Mean for the IjTight . . 39
Annual Mean .... 47
Wind.
SW
NE
SW
ANNUAL RESULTS.
Barometer.
Highest . December 23, 30.61
Lowest . October 19, 28.81
*. '.-OS
Annual Mean
29.93
Wind.
NE
SW
Wind. Times.
N
NE
E
SE
? 54
? 40
? 13
? _5S
105
Wind. Times
S ? 42]
SW ? 95
W ? 38'
NW ? _6g|
241
1G5|
Total 406!
Weather.Days.
Fine . . 179
Dloudy 44
Wet . . 143
366

				

